# Oral Curcumin With Piperine as Adjuvant Therapy for the Treatment of COVID-19: A Randomized Clinical Trial

**DOI:** 10.3389/fphar.2021.669362

**Published:** 2021-05-28

**Authors:** Kirti S Pawar, Rahul N Mastud, Satheesh K Pawar, Samragni S Pawar, Rahul R Bhoite, Ramesh R Bhoite, Meenal V Kulkarni, Aditi R Deshpande

**Affiliations:** ^1^ Giriraj Hospital and Intensive Care unit, Baramati, India; ^2^ Siddhivinayak Ultrasound Research Center, Baramati, India; ^3^ HBT Medical College and Dr R N Cooper Municipal General Hospital, Mumbai, India; ^4^ Medstar Good Samaritan Hospital, Baltimore, MD, United States; ^5^ Department of Preventive and Social Medicine, N K P Salve Medical College Nagpur, Nagpur, India; ^6^ Freelance Statistician, Nagpur, India

**Keywords:** curcumin, COVID-19, adjuvant therapy, traditional medicine, anti-inflammatory, post-covid thromboembolic episodes

## Abstract

**Background:** Coronavirus disease-2019 (COVID-19) has a wide range of pathophysiological effects. Curcumin, an active constituent of Curcuma longa (turmeric), has several properties, including anti-inflammatory, antioxidant, antiviral, anti-thrombotic, and anti-proliferative effects, which make it a promising candidate for the symptomatic treatment of COVID-19.

**Objective:** We aimed to determine the effects of curcumin administered with piperine (to optimize absorption) on symptoms in patients with COVID-19 in a double-blind, randomized, controlled trial at a 30-bed dedicated COVID Health Center (DCHC) in Maharashtra, India.

**Methods:** In addition to conventional COVID-19 treatment, patients in the control group received a dose of probiotics twice a day, and patients in the study group received curcumin (525 mg) with piperine (2.5 mg) in tablet form twice a day. The effects of curcumin/piperine treatment on primary and secondary outcomes were assessed for the duration of hospitalization.

**Results:** Patients with mild, moderate, and severe symptoms who received curcumin/piperine treatment showed early symptomatic recovery (fever, cough, sore throat, and breathlessness), less deterioration, fewer red flag signs, better ability to maintain oxygen saturation above 94% on room air, and better clinical outcomes compared to patients of the control group. Furthermore, curcumin/piperine treatment appeared to reduce the duration of hospitalization in patients with moderate to severe symptoms, and fewer deaths were observed in the curcumin/piperine treatment group.

**Conclusions:** Administration of oral curcumin with piperine as an adjuvant symptomatic therapy in COVID-19 treatment could substantially reduce morbidity and mortality, and ease the logistical and supply-related burdens on the healthcare system. Curcumin could be a safe and natural therapeutic option to prevent Post-Covid thromboembolic events.

**Clinicaltrials.gov identifier:** CTRI/2020/05/025482

## Introduction

The ongoing Coronavirus Disease 2019 (COVID-19) pandemic, caused by the novel coronavirus SARS-CoV-2, has had devastating medical, social, and economic impacts worldwide. Patients with COVID-19 can present with acute symptoms of fever, dyspnea, and pneumonia (Huang et al., 2020). Atypical symptoms like skin rashes, thrombosis, and anosmia, and acute respiratory distress syndrome (ARDS) and multi-system failure due to cytokine release syndrome (CRS) have also been reported. With the increase in cases and the lack of a specialized cure, therapeutic approaches have been developed globally to counter specific aspects of the disease. The protocol provided by the Indian COVID-19 Taskforce includes the administration of the following: antimicrobials, such as doxycycline, remdesivir, pavipiravir, oseltamivir, and ivermectin, to reduce viral load; anti-inflammatory medications, such as hydroxychloroquine, tocilizumab, and itolizumab, to reduce the effect of the cytokine storm; blood thinners, such as unfractionated heparin and low molecular weight heparin, to prevent coagulopathy and thromboembolic complications; anti-inflammatory and immunosuppressant drugs, such as methyl prednisolone and dexamethasone, to reduce the intensity of cytokine storm and lung fibrosis; and nutritional supplements to boost immunity.

Curcumin, the bioactive ingredient of *Curcuma longa* (turmeric) has a wide range of therapeutic effects (Loeber and Buechner 1748; Vogel and Pelletier, 1815; Oppenheimer, 1937; Keihanian et al., 2018) that make it an excellent candidate for use as adjuvant therapy in the treatment of patients with COVID-19. Curcumin has potential antiviral effects, including protein binding affinity towards SARS-CoV-2 proteins, which is comparable to that of drugs such as hydroxychloroquine that have been considered for clinical trials in the treatment of COVID-19 (Maurya et al., 2020). Moreover, curcumin exhibits anti-inflammatory (Gupta et al., 2013), antioxidant, anti-bacterial, antiviral, antifungal, anti-thrombotic, anti-proliferative, hypoglycemic, anticarcinogenic, neuroprotective, and cardioprotective properties (Aggarwal and Harikumar, 2009; Keihanian et al., 2018) and modulates several signaling molecules, including pro-inflammatory cytokines, apoptotic proteins, NF–κB, cyclooxygenase-2, 5-LOX, STAT3, C-reactive protein, prostaglandin E2, prostate-specific antigen, adhesion molecules, phosphorylase kinase, transforming growth factor-β, triglyceride, ET-1, creatinine, HO-1, AST, and ALT (Shanmugam et al., 2015). Curcumin inhibits thrombin and FXa and reduces blood viscosity; it could therefore alleviate COVID coagulopathy and thereby increase survival rates (Keihanian et al., 2018). Preclinical studies have shown that curcumin effectively inhibits viral infection, alleviates the severity of lung injury by offsetting the cytokine storm, and inhibits subsequent fibrosis (Liu and Ying, 2020). Clinical trials on the use of curcumin in the treatment of skin, eye, CNS, respiratory, cardiovascular, gastrointestinal, urogenital, inflammatory, and metabolic disorders have been reported (Salehi et al., 2019). Further, curcuminoids have been approved by the US Food and Drug Administration (FDA) as “Generally Recognized As Safe” (GRAS) and have good tolerability and safety profiles (Hewlings and Kalman, 2017), and few adverse side-effects have been reported with the long and short-term use of curcumin.

Curcumin exhibits very poor bioavailability, with very low or undetectable concentrations in blood and extraintestinal tissues, likely due to poor absorption, rapid metabolism, chemical instability, and rapid systemic elimination (Anand et al., 2007; Toden and Goel, 2017). Piperine, a Bio-enhancer, considerably improves the absorption of curcumin (Shoba et al., 1998; Sharma et al., 2010; Suresh and Srinivasan, 2010) by up to 2,000-fold (Han, 2011). We conducted a pilot study to determine the effects of curcumin and piperine in patients with COVID-19 and observed favorable trends. We, therefore, conducted the present study to evaluate the efficacy of oral curcumin with piperine as an adjuvant symptomatic therapy for the treatment of COVID-19.

## Methods

### Study Design

We performed a double-blind, randomized, controlled trial with probiotic control at a 30-bed Dedicated COVID Health Center (DCHC) in Maharashtra, India. Because there is no institutional ethics committee, ethical approval was obtained from the Royal Pune Independent Ethics Committee. The study is registered at http://www.clinicaltrials.gov with the identifier CTRI/2020/05/025482, and the study protocol is included therein.

### Participants

We recruited symptomatic adult patients (over 18 years of age) with positive COVID-19 antigen tests from July to September 2020. Exclusion criteria were as follows: a critical group of patients in whom intravenous drugs were the most effective on admission, patients who required ventilator support within 24 h of admission (presented with Acute Respiratory Distress Syndrome), patients who were pregnant or breast-feeding, patients receiving coumarin anticoagulants, patients with known allergic reactions to curcumin and its products, and asymptomatic patients. The relatives of the recruited patients or their next of kin provided written informed consent for participation. Consent forms were available to patients in English and two local languages.

### Randomization and Masking

The participants were randomly assigned to control and study groups, which each included 70 patients. Patients in both groups received the conventional COVID-19 treatment defined by the Maharashtra state COVID-19 task. In addition, patients in the control group received probiotics, and patients in the study group received curcumin with piperine in similar capsules.

A programmer who had no role in recruitment, treatment, or assessment of patients independently generated the randomization schedule and allocation sequence. The minimum sample size calculated for each group was 60. However, our pilot study showed that a proportion of patients with COVID-19 with mild (SpO_2_ > 94%), moderate (SpO_2_ between 90 and 94 with pneumonitis), and severe (SpO_2_ < 90%) symptoms was 6:5:3. To avoid selection bias and account for possible dropouts, we chose a sample size of 70, with similar proportions of patients of each subgroup (30 patients with mild symptoms, 25 with moderate symptoms, and 15 with severe symptoms in the control and study groups). The allocation sequence for each subgroup (mild, moderate, and severe) was created using a randomization application. The schedule was not disclosed until the trial was completed.

Hospital staff who administered the treatments to the patients were provided with three sets (Mild: 60, Moderate:50, and Severe:30) of randomly assigned opaque envelopes, each marked with a unique number. Half the envelopes of each set contained probiotics, and the other half contained the curcumin formulation. Because curcumin has a good safety profile, we did not expect adverse effects, and an interim analysis was not planned. After obtaining consent and initial clinical examination, the treating physician assigned patients into the mild, moderate, or severe subgroups, based on their clinical condition. An envelope was then picked from the corresponding subgroup set at random, without replacement, and added to the conventional drug regimen administered. The trial was performed until the completion of treatment in the patients of each subgroup.

Participants, hospital staff who administered the treatment, and researchers assessing outcomes and analyzing data were masked to group assignment.

### Procedures

Patient demographics are shown in Table 1. All patients included in this study tested positive in a COVID-19 antigen test and were admitted to the rural hospital at Rui, Baramati in Maharashtra, India, which is a dedicated COVID health center. Patients were assigned into the following subgroups based on their clinical condition during admission: mild symptoms (SpO_2_ > 94% on room air), moderate symptoms (SpO_2_ between 90 and 94% on room air, with pneumonitis), and severe symptoms (SpO_2_ < 90% on room air). Patients with mild and moderate symptoms were admitted to wards, and those with severe symptoms were admitted to the intensive care unit.

**TABLE 1:** Patient demographics.

Demographics		Mild		Moderate		Severe		All combined	
		C3 group	Non C3 group	C3 group	Non C3 group	C3 group	Non C3 group	C3 group	Non C3 group
Sample size (*n*)		30	30	25	25	15	15	70	70
Sex	Male	24 (80.0%)	20 (66.7%)	14 (56.0%)	23 (92.0%)	7 (46.7%)	11 (73.3%)	45 (64.3%)	54 (77.1%)
	Female	6 (20.0%)	10 (33.3%)	11 (44.0%)	2 (8.0%)	8 (53.3%)	4 (26.7%)	25 (35.7%)	16 (22.9%)
Risk fact	Age >60	3 (10.0%)	9 (30.0%)	10 (40.0%)	6 (24.0%)	5 (33.3%)	5 (33.3%)	18 (25.7%)	20 (28.6%)
Pneumonia	Unilateral	0 (0.0%)	0 (0.0%)	4 (16.0%)	1 (4.0%)	0 (0.0%)	1 (6.7%)	4 (5.7%)	2 (2.9%)
	Bilateral	0 (0.0%)	0 (0.0%)	20 (80.0%)	24 (96.0%)	15 (100.0%)	14 (93.3%)	35 (50.0%)	38 (54.3%)
Age in years	Min	18	25	27	25	22	40	18	25
	Max	60	77	85	84	80	80	85	84

**FIGURE 1:**
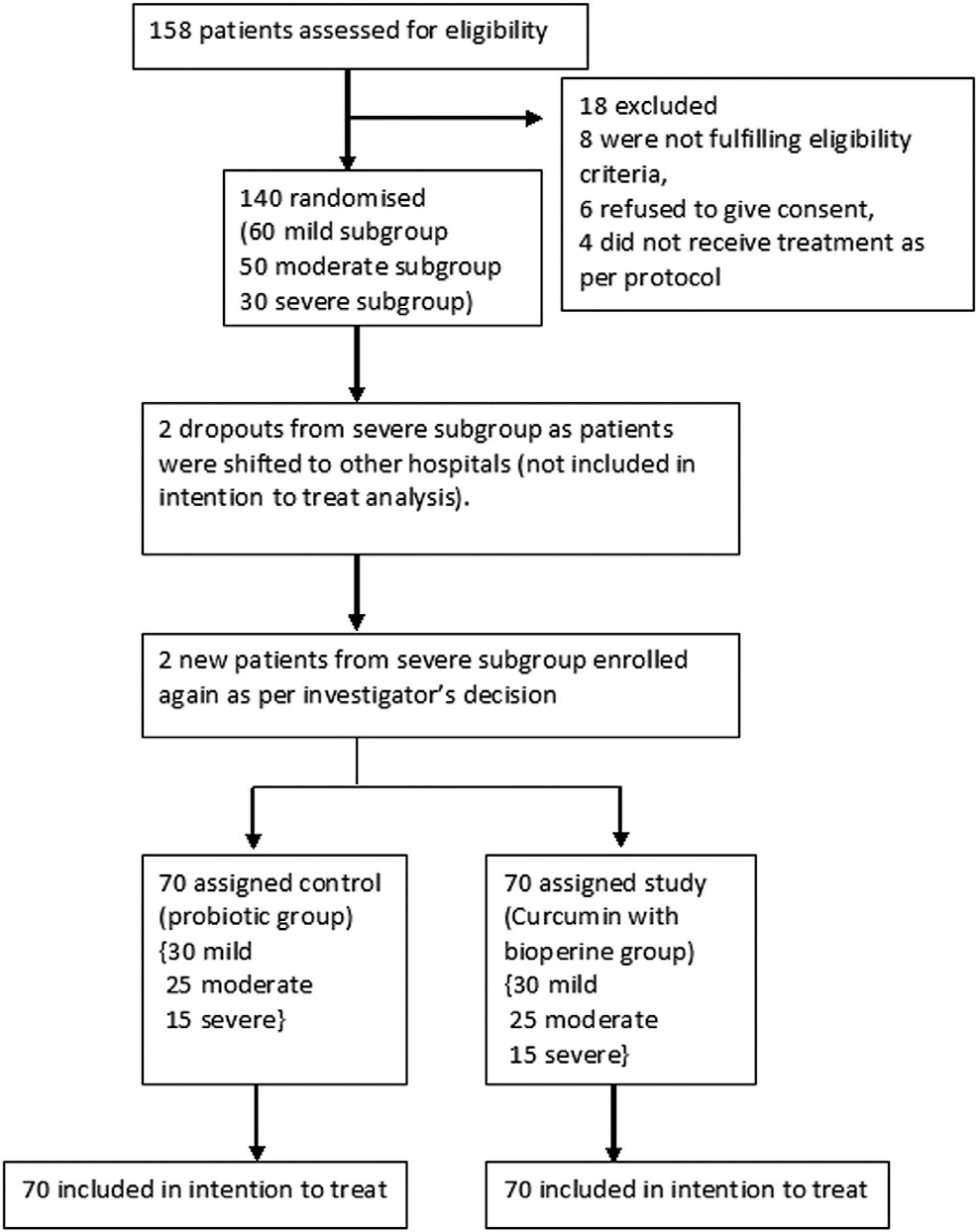
CONSORT flow diagram for this study.

The study group comprising patients who received curcumin and piperine was designated as the C3 group, and the control group with patients who received probiotics was designated as the non-C3 group. All patients received treatment based on the clinical management protocol: Covid-19 (version 5). In addition, the control group received a probiotic capsule (Nutrolin B Plus, which contains lactic acid *Bacillus* and Vitamin B; Ciplamed), and the study group received USFDA-approved Curcumin C3 Complex^®^ (SamiDirect, India) dietary supplement tablets containing 525 mg Tab. Curcumin (diferuloylmethane; 525 mg) with 2.5 mg Bioperine^®^ (2.5 mg; SamiDirect) twice a day for 14 days from the day of admission Figure 1.

Blood testing for complete blood count, C-reactive protein, and D-Dimer was performed on admission and subsequently if required. Temperature, pulse, respiratory rate, and SpO_2_ were monitored every 4 h in patients of the mild and moderate subgroups, and continuously in patients of the severe subgroup. The following outcomes were measured in the control and study groups for the duration of stay in the hospital.

#### Outcomes

The primary outcomes assessed are laid out below.

PO 1: Number of days required for remission of symptoms (DRR): fever, cough, sore throat, breathlessness.

PO 2: Patient showed the following red flag signs.

1. Absolute Neutrophil to Absolute Lymphocyte ratio >3.5 
2. P:F ratio <300.
3. Rising CRP, Ferritin, D-dimer, LDH, and Triglycerides.
4. Troponin I Positive and Positive CK-MB.

PO 3: Patient maintained SpO_2_ above 94% on room air throughout hospital stay.

PO 4: Number of days patient could not maintain SpO_2_ above 94% on room air.

PO 5: Patient required Oxygen therapy.

PO 6: Number of days patient was on oxygen support.

PO 7: Patient required oxygen therapy with high flow nasal cannula (HFNC).

PO 8: Patient failed to maintain SpO_2_ >88% despite oxygenation.

PO 9: Patient required

a. intubation
b. mechanical ventilation (noninvasive/invasive).
MV- (NI) Non invasive.
MV- (I) Invasive.

PO 10: Laboratory parameters: a. CRP b. D-dimer c. Hb d. LDH e. Neutrophils/Lymphocytes (N/L ratio) >3.5 or N/L < 3.5.

PO 11: Chest X Ray abnormalities.

PO 12: Patient required use of the COVID awake repositioning/proning protocol (CARP).

PO 13: Patient required LMW Heparin.

PO 14: Patient required remdesivir.

PO 15: Patient required cytokine storm treatment.

The secondary outcomes assessed were:

SO 1: Duration of mechanical ventilator assistance.

SO 2: Duration of hospitalization: a. fewer than 10 days; b. 10–14 days; c. more than 14 days.

SO 3: Presence of thromboembolic events such as MI, stroke, pulmonary embolism, or DVT during hospital stay.

SO 4: Death related to SARS CoV2 and causes of mortality.

Adverse drug reactions that we monitored for were: ADR1: nausea, vomiting, abdominal discomfort, ADR2: skin rash, ADR3: burning micturition, ADR4: excessive bleeding, hematuria.

Patients were discharged when they remained asymptomatic for >48 h.

### Statistical Analysis

Based on the size of our study population and the results of the pilot study, we estimated that our trial had about 90% power to detect, at a 5% significance level, a 30% difference in the proportion of patients who required oxygen support for recovery (55% control group *vs*. 25% study group). The minimum sample size calculated for each group was 60; we chose a sample size of 70 for each group to account for possible drop-outs. However, there were no drop-outs during this study. Data analysis was performed using SPSS version 20.0 and Microsoft Office Excel 2007. The demographic variables were tabulated using frequency distributions. Continuous variables were analyzed using two-way ANOVA for the two groups and the three levels of severity. We also performed post hoc tests for significant results of two-way ANOVA for severity. Binary/Categorical variables were analyzed using the odds ratio.

## Results

Baseline characteristics of clinical presentation in the two treatment groups are shown in Table 1.

### Primary Outcomes

As shown in Supplementary Tables S1–S3, in the mild, moderate, and severe subgroups, compared to the patients of the non-C3 group, patients of the C3 group showed early symptomatic recovery (of fever, cough, sore throat, breathlessness**)**, less deterioration over the period of hospital stay, lower incidence of red flag signs, and better ability to maintain oxygen saturation above 94% on room air throughout the hospital stay. Further, patients of the C3 group could maintain SpO_2_ above 94% on room air for more days, and fewer patients of the C3 group required oxygen therapy with or without HFNO, failed to maintain SpO_2_ >88% despite oxygenation, or required intubation and mechanical ventilation to maintain SpO_2_ above 88%. Although deterioration in laboratory parameters was observed in patients from both groups, the rise in D-Dimer levels was less severe in the C3 Group than in the non-C3 group.

The Neutrophil/lymphocyte ratio was >3.5 in both groups, and differences between the moderate and severe subgroups were not significant. 3/30 patients from the mild C3 subgroup and 7/30 from the non-C3 subgroup showed the development of pneumonitis on chest X-rays during hospitalization. All patients in the moderate and severe subgroups of the C3 and non-C3 groups presented with bilateral pneumonitis, except one patient in the moderate C3 subgroup, who had unilateral pneumonitis.

Fewer patients in the study group required the administration of the COVID awakening protocol, remdesivir, low molecular weight heparin, or unfractionated heparin in addition to oral doxycycline, favipiravir, steroids, and nutritional supplements. None of the patients in the moderate C3 subgroup required tocilizumab injections, while 5/25 patients in the Moderate Non-C3 subgroup did. 2/15 patients from the severe C3 subgroup and 4/15 from the severe Non-C3 subgroup required and received tocilizumab per protocol. Unfortunately, none of these patients survived.

### Secondary Outcomes

As shown in , the duration of hospitalization was almost the same in the C3 and non-C3 mild subgroups; however, it was significantly lower in the moderate and severe C3 subgroups than in the corresponding non-C3 subgroups. Fewer patients required mechanical ventilator support and suffered thromboembolic episodes in the C3 group than in the non-C3 group. Oral aspirin was not given to any of these patients.

There were no deaths in the mild and moderate C3 subgroups, while there was 1 death out of 30 in the mild non-C3 subgroup and 5 deaths out of 25 in the moderate non-C3 subgroup. There were fewer deaths (2/15) in the severe C3 subgroup than in the severe non-C3 subgroup (5/15).

## Discussion

Our results indicate that the administration of oral curcumin with piperine as an adjuvant therapy could significantly improve the effects of the COVID-19 treatment protocol. Administration of oral curcumin with piperine reduced the days of remission of symptoms (*viz*. fever, cough, sore throat, and breathlessness**)**, oxygen requirement, and the need for mechanical ventilation and remdesivir injections as well as secondary outcomes, such as days of mechanical ventilation and hospitalization, and number of thromboembolic episodes.

The known pathophysiology of COVID-19 mainly involves life-threatening inflammatory reactions, cytokine storms and coagulopathy. Inflammation is the first observed insult in this disease. Viral infection and inflammation-induced hemoconcentration and difficulty in the exchange of oxygen and carbon dioxide (apparently mimicking polycythemia vera) could be a prominent cause of hypoxia. Curcumin has anti-inflammatory activity and exerts antithrombotic effects *via* the inhibition of thrombin and FXa (Kim et al., 2012). It can reduce the viscosity of blood and thereby the risk of developing blood clots and the associated complications. Therefore, curcumin has great potential as an anti-inflammatory drug in the treatment of COVID-19. In our study, curcumin, when combined with antivirals and other blood thinners, showed good efficacy. Like oral aspirin and clopidogrel, which are used as an adjuvant therapy with thrombolytic agents for the treatment of myocardial infarction, oral curcumin would likely help alleviate COVID coagulopathy when administered with or without heparin. Moreover, the antibacterial and antifungal effects of curcumin could play a role in preventing secondary infections and therefore promote early recovery.

The known risk factors for COVID-19 include age >60 years, diabetes (Zhang et al., 2013; Poolsup et al., 2019), hypertension (Leong, 2018), cardiac disease (Wongcharoen and Phrommintikul 2009), chronic lung disease (Venkatesan et al., 2007; Biswas and Rahman, 2008; Moriyuki et al., 2010), cerebrovascular disease (Ovbiagele, 2008), chronic kidney disease (Ghosh et al., 2014; de Almeida Alvarenga et al., 2018), immunosuppression (Sharma et al., 2007; Mollazadeh et al., 2019) and cancer (Ravindran et al., 2009). Curcumin is an effective adjuvant in the treatment of each of these conditions. Further, curcumin is typically not associated with adverse effects, such as oxygen toxicity and heparin-induced thrombocytopenia, and is safe for long-term use (Gupta et al., 2013).

Endotoxins released by microorganisms activate macrophages and neutrophils to produce inflammatory cytokines and eicosanoids such as thromboxane A2 (Conti et al., 2020); anti-inflammatory medications such as COX-2 inhibitors could cause an imbalance prostaglandin production. Greater affinity for the COX-2 isoform could cause accumulation of thromboxane, a pro-aggregatory and vasoconstrictive prostaglandin produced from the COX-1 isoform, and therefore increase cardiovascular risk in patients with COVID-19. In this context, curcumin is unique because of its anti-inflammatory and antithrombotic effects. In patients with COVID-19 who exhibit long-term thromboembolic complications, curcumin treatment could reduce thromboembolic complications with lower bleeding risk, when low molecular weight heparin or unfractionated heparin treatment is stopped or in the absence of heparin treatment. Several preclinical studies have shown that curcumin could prevent cytokine storms (Shanmugam et al., 2015) and COVID coagulopathy (Singh, 2020; Suresh, 2020), and function as an antiviral drug (Zahedipour et al., 2020). To our knowledge, our study is the first to describe the efficacy of orally administered curcumin with piperine in the symptomatic treatment of COVID-19.

Our study had some limitations: we attempted to measure activated partial thromboplastin time (aPTT) and prothrombin time (PT) in all the patients but could perform these tests only in a small number of patients who were receiving low molecular weight heparin, due to limited resources. None of these patients showed an increase in INR over the control value. Retrospectively, we found that patients who received curcumin had near-normal INR, and patients who did not receive curcumin had values lower than the control.

Since self-management of symptoms is also widespread in the current pandemic, future studies on the use of curcumin for self-management of COVID-19 symptoms could be of interest.

## Conclusion

Orally administered curcumin with piperine could play a multifaceted role in the treatment of COVID-19. The anti-inflammatory and anti-thrombotic properties of curcumin could expedite the recovery of COVID-19 patients, and its antiviral, antibacterial, and antifungal properties could prevent superadded or secondary infections. Our results suggest that the use of orally administered curcumin with piperine as adjuvant therapy in COVID-19 treatment could substantially reduce morbidity and mortality, reduces treatment costs, and decrease logistical burden healthcare systems. Dose-escalating studies have indicated the safety of curcumin over 3 months. Hence, Curcumin can be a safe and natural therapeutic option to prevent Post-Covid thromboembolic events.

## Data Availability

The original contributions presented in the study are included in the article/Supplementary Material, further inquiries can be directed to the corresponding author.
