# Supplementary material for: Oral Curcumin With Piperine as Adjuvant Therapy for the Treatment of COVID-19: A Randomized Clinical Trial
Source: Front Pharmacol. 2021 May 28;12:669362. doi: 10.3389/fphar.2021.669362 (PMC8193734; doi:10.3389/fphar.2021.669362)
Supplement: Supplementary file 1 [file Table1.pdf]

Supplementary Table 1: Mild subgroup: Statistical Analysis of Primary and Secondary outcomes.

Mild						
Parameters			C3 Group	Non C3 Group	Odds Ratio (95% CI)	Significance
Sample Size (n)			30	30		
Sex	Male		24 (80.0%)	20 (66.7%)		
	Female		6 (20.0%)	10 (33.3%)		
Risk Factor	Age >60		3 (10.0%)	9 (30.0%)	0.26 (0.06, 1.08)	Not Significant
	Unilateral pneumonia		0 (0.0%)	0 (0.0%)	Not Applicable	
	Bilateral pneumonia		0 (0.0%)	0 (0.0%)	Not Applicable	
Fever			16 (53.3%)	12 (40.0%)	1.71 (0.62, 4.77)	Not Significant
Cold			9 (30.0%)	9 (30.0%)	1.00 (0.33, 3.02)	Not Significant
Cough			14 (46.7%)	19 (63.3%)	0.51 (0.18, 1.42)	Not Significant
Sore throat			5 (16.7%)	6 (20.0%)	0.80 (0.22, 2.97)	Not Significant
Sneezing			0 (0.0%)	1 (3.3%)	Not Applicable	
Runny nose			0 (0.0%)	2 (6.7%)	Not Applicable	
Shortness of breath			7 (23.3%)	7 (23.3%)	1.00 (0.30, 3.31)	Not Significant
Headache			3 (10.0%)	4 (13.3%)	0.72 (0.15, 3.54)	Not Significant
Diarrhoea			1 (3.3%)	3 (10.0%)	0.31 (0.03, 3.17)	Not Significant
Myalgia			4 (13.3%)	7 (23.3%)	0.51 (0.13, 1.95)	Not Significant
Fatigue			2 (6.7%)	6 (20.0%)	0.29 (0.05, 1.55)	Not Significant
Loss of taste			0 (0.0%)	0 (0.0%)	Not Applicable	
Symptoms<6d			30 (100%)	27 (90.0%)	Not Applicable	
Symptoms>6d			0 (0.0%)	3 (10.0%)	Not Applicable	
PO2	Patient showed red flag signs	Yes	3 (10.0%)	7 (23.3%)	0.37 (0.08, 1.58)	Not Significant
		No	27 (90.0%)	20 (66.7%)	4.50 (1.09, 18.50)	Significant
PO3	Patient maintained SpO2 above 94% on room air throughout the stay	Yes	28 (93.3%)	19 (63.3%)	8.11 (1.61, 40.77)	Significant
		No	2 (6.7%)	10 (33.3%)	0.14 (0.03, 0.72)	Significant
PO4	Number of days patient could not maintain SpO2 above 94% on room air	No	28 (93.3%)	21 (70.0%)	6.00 (1.17, 30.73)	Significant
PO5	Patient required Oxygen therapy	Yes	2 (6.7%)	10 (33.3%)	0.14 (0.03, 0.72)	Significant
		No	28 (93.3%)	21 (70.0%)	6.00 (1.17, 30.73)	Significant
PO7	Patient required Oxygen therapy with high flow nasal cannula (HFNC)	Yes	0 (0.0%)	1 (3.3%)	Not Applicable	
		No	30 (100%)	28 (93.3%)	Not Applicable	
PO8	Patient failed to maintain SpO2 more than 88 % despite of oxygenation	Yes	0 (0.0%)	1 (3.3%)	Not Applicable	
		No	30 (100%)	29 (96.7%)	Not Applicable	
PO9	intubation	Yes	0 (0.0%)	0 (0.0%)	Not Applicable	
		No	30 (100%)	30 (100%)	Not Applicable	
	mechanical ventilation (noninvasive / invasive)	MV-NI	0 (0.0%)	1 (3.3%)	Not Applicable	
		MV-I	0 (0.0%)	0 (0.0%)	Not Applicable	
PO10	CRP rise		3 (10.0%)	4 (13.3%)	0.72 (0.15, 3.54)	Not Significant
	CRP decline		0 (0.0%)	0 (0.0%)	Not Applicable	
	CRP approximately same		27 (90.0%)	15 (50.0%)	9.00 (2.24, 36.17)	Significant
	D-Dimer rise		0 (0.0%)	0 (0.0%)	Not Applicable	
	D-Dimer decline		0 (0.0%)	0 (0.0%)	Not Applicable	
	D-Dimer approximately same		29 (96.7%)	19 (63.3%)	16.79 (2.00, 140.90)	Significant
	N/L>3.5		4 (13.3%)	3 (10.0%)	1.38 (0.28, 6.80)	Not Significant
	N/L<3.5		25 (83.3%)	15 (50.0%)	5.00 (1.51, 16.56)	Significant
PO11	Chest X Ray abnormalities	Yes	3 (10.0%)	8 (26.7%)	0.31 (0.07, 1.29)	Not Significant
		No	27 (90.0%)	22 (73.3%)	3.27 (0.77, 13.83)	Not Significant
PO12	Patient required COVID CARP protocol	Yes	0 (0.0%)	7 (23.3%)	Not Applicable	
		No	30 (100%)	23 (76.7%)	Not Applicable	
PO13	Patient required LMW Heparin	Yes	2 (6.7%)	4 (13.3%)	0.46 (0.08, 2.75)	Not Significant
		No	28 (93.3%)	26 (86.7%)	2.15 (0.36, 12.76)	Not Significant
PO14	Patient required Remdesivir	Yes	1 (3.3%)	6 (20.0%)	0.14 (0.02, 1.23)	Not Significant
		No	29 (96.7%)	24 (80.0%)	7.25 (0.82, 64.46)	Not Significant
PO15	Patient required cytokine storm treatment eg. Tocilizumab	Yes	0 (0.0%)	0 (0.0%)	Not Applicable	
		No	30 (100%)	30 (100%)	Not Applicable	
SO2	Duration of hospitalisation	<10 days	30 (100%)	25 (83.3%)	Not Applicable	
		10-14 day	0 (0.0%)	4 (13.3%)	Not Applicable	
		>14days	0 (0.0%)	1 (3.3%)	Not Applicable	
SO3	Thromboembolic events	Yes	0 (0.0%)	1 (3.3%)	Not Applicable	
		No	30 (100%)	29 (96.7%)	Not Applicable	
	Pulmonary fibrosis	Yes	0 (0.0%)	2 (6.7%)	Not Applicable	
		No	30 (100%)	26 (86.7%)	Not Applicable	
SO4	Death related to SARS CoV2 and causes of mortality	Yes	0 (0.0%)	1 (3.3%)	Not Applicable	
		No	30 (100%)	29 (96.7%)	Not Applicable	
ADR1	Nausea, Vomiting, Abdominal discomfort	Yes	0 (0.0%)	0 (0.0%)	Not Applicable	
		No	30 (100%)	30 (100%)	Not Applicable	
ADR2	Skin Rash	Yes	0 (0.0%)	0 (0.0%)	Not Applicable	
		No	30 (100%)	30 (100%)	Not Applicable	
ADR3	Burning micturition	Yes	0 (0.0%)	0 (0.0%)	Not Applicable	
		No	30 (100%)	30 (100%)	Not Applicable	
ADR4	Excessive bleeding	Yes	0 (0.0%)	0 (0.0%)	Not Applicable	
		No	30 (100%)	30 (100%)	Not Applicable	
PO - Primary Outcome; SO - Secondary Outcome; ADR - Adverse drug reaction						
If 95% confidence interval for odds ratio contains value 1, then the parameter is not significant at 5% level.						
