# Supplementary material for: Oral Curcumin With Piperine as Adjuvant Therapy for the Treatment of COVID-19: A Randomized Clinical Trial
Source: Front Pharmacol. 2021 May 28;12:669362. doi: 10.3389/fphar.2021.669362 (PMC8193734; doi:10.3389/fphar.2021.669362)
Supplement: Supplementary file 2 [file Table2.pdf]

Supplementary Table 2: Moderate subgroup: Statistical Analysis of Primary and Secondary outcomes.

Moderate						
Parameters			C3 Group	Non C3 Group	Odds Ratio (95% CI)	Significance
Sample Size (n)			25	25		
Sex	Male		14 (56.0%)	23 (92.0%)		
	Female		11 (44.0%)	2 (8.0%)		
Risk Factor	Age >60		10 (40.0%)	6 (24.0%)	2.11 (0.62, 7.13)	Not Significant
	Unilateral pneumonia		4 (16.0%)	1 (4.0%)	4.57 (0.47, 44.17)	Not Significant
	Bilateral pneumonia		20 (80.0%)	24 (96.0%)	0.17 (0.02, 1.55)	Not Significant
Fever			20 (80.0%)	17 (68.0%)	1.88 (0.52, 6.84)	Not Significant
Cold			11 (44.0%)	8 (32.0%)	1.67 (0.53, 5.29)	Not Significant
Cough			18 (72.0%)	16 (64.0%)	1.45 (0.44, 4.78)	Not Significant
Sore throat			3 (12.0%)	1 (4.0%)	3.27 (0.32, 33.84)	Not Significant
Sneezing			0 (0.0%)	0 (0.0%)	Not Applicable	
Runny nose			0 (0.0%)	1 (4.0%)	Not Applicable	
Shortness of breath			6 (24.0%)	15 (60.0%)	0.21 (0.06, 0.71)	Significant
Headache			5 (20.0%)	1 (4.0%)	6.00 (0.65, 55.66)	Not Significant
Diarrhoea			1 (4.0%)	1 (4.0%)	1.00 (0.06, 16.93)	Not Significant
Myalgia			6 (24.0%)	2 (8.0%)	3.63 (0.66, 20.12)	Not Significant
Fatigue			7 (28.0%)	2 (8.0%)	4.47 (0.83, 24.19)	Not Significant
Loss of taste			1 (4.0%)	0 (0.0%)	Not Applicable	
Symptoms<6d			22 (88.0%)	21 (84.0%)	1.40 (0.28, 7.00)	Not Significant
Symptoms>6d			3 (12.0%)	3 (12.0%)	1.00 (0.18, 5.51)	Not Significant
PO2	Patient showed red flag signs	Yes	13 (52.0%)	25 (100.0%)	Not Applicable	
		No	13 (52.0%)	0 (0.0%)	Not Applicable	
PO3	Patient maintained SpO2 above 94% on room air throughout the stay	Yes	14 (56.0%)	2 (8.0%)	14.64 (2.82, 75.96)	Significant
		No	12 (48.0%)	23 (92.0%)	0.08 (0.02, 0.42)	Significant
PO4	Number of days patient could not maintain SpO2 above 94% on room air	No	5 (20.0%)	1 (4.0%)	6.00 (0.65, 55.66)	Not Significant
PO5	Patient required Oxygen therapy	Yes	13 (52.0%)	25 (100.0%)	Not Applicable	
		No	12 (48.0%)	0 (0.0%)	Not Applicable	
PO7	Patient required Oxygen therapy with high flow nasal cannula (HFNC)	Yes	0 (0.0%)	9 (36.0%)	Not Applicable	
		No	25 (100.0%)	16 (64.0%)	Not Applicable	
PO8	Patient failed to maintain SpO2 more than 88 % despite of oxygenation	Yes	0 (0.0%)	6 (24.0%)	Not Applicable	
		No	25 (100.0%)	19 (76.0%)	Not Applicable	
PO9	intubation	Yes	0 (0.0%)	0 (0.0%)	Not Applicable	
		No	15 (60.0%)	17 (68.0%)	0.71 (0.22, 2.25)	Not Significant
	mechanical ventilation (noninvasive / invasive)	MV-NI	0 (0.0%)	6 (24.0%)	Not Applicable	
		MV-I	0 (0.0%)	0 (0.0%)	Not Applicable	
PO10	CRP rise		14 (56.0%)	24 (96.0%)	0.05 (0.01, 0.46)	Significant
	CRP decline		0 (0.0%)	0 (0.0%)	Not Applicable	
	CRP approximately same		6 (24.0%)	0 (0.0%)	Not Applicable	
	D-Dimer rise		3 (12.0%)	6 (24.0%)	0.43 (0.09, 1.97)	Not Significant
	D-Dimer decline		0 (0.0%)	0 (0.0%)	Not Applicable	
	D-Dimer approximately same		7 (28.0%)	1 (4.0%)	9.33 (1.05, 82.78)	Significant
	N/L>3.5		9 (36.0%)	11 (44.0%)	0.72 (0.23, 2.23)	Not Significant
	N/L<3.5		6 (24.0%)	6 (24.0%)	1.00 (0.27, 3.66)	Not Significant
PO11	Chest X Ray abnormalities	Yes	24 (96.0%)	25 (100.0%)	Not Applicable	
		No	1 (4.0%)	0 (0.0%)	Not Applicable	
PO12	Patient required COVID CARP protocol	Yes	11 (44.0%)	22 (88.0%)	0.11 (0.03, 0.45)	Significant
		No	14 (56.0%)	3 (12.0%)	9.33 (2.21, 39.46)	Significant
PO13	Patient required LMW Heparin	Yes	14 (56.0%)	24 (96.0%)	0.05 (0.01, 0.46)	Significant
		No	11 (44.0%)	1 (4.0%)	18.86 (2.20, 161.99)	Significant
PO14	Patient required Remdesivir	Yes	7 (28.0%)	22 (88.0%)	0.05 (0.01, 0.24)	Significant
		No	18 (72.0%)	3 (12.0%)	18.86 (4.25, 83.59)	Significant
PO15	Patient required cytokine storm treatment eg. Tocilizumab	Yes	0 (0.0%)	5 (20.0%)	Not Applicable	
		No	25 (100.0%)	20 (80.0%)	Not Applicable	
SO2	Duration of hospitalisation	<10 days	25 (100.0%)	11 (44.0%)	Not Applicable	
		10-14 day	0 (0.0%)	10 (40.0%)	Not Applicable	
		>14days	0 (0.0%)	4 (16.0%)	Not Applicable	
SO3	Thromboembolic events	Yes	0 (0.0%)	4 (16.0%)	Not Applicable	
		No	25 (100.0%)	21 (84.0%)	Not Applicable	
	Pulmonary fibrosis	Yes	0 (0.0%)	12 (48.0%)	Not Applicable	
		No	25 (100.0%)	13 (52.0%)	Not Applicable	
SO4	Death related to SARS CoV2 and causes of mortality	Yes	0 (0.0%)	5 (20.0%)	Not Applicable	
		No	25 (100.0%)	20 (80.0%)	Not Applicable	
ADR1	Nausea, Vomiting, Abdominal discomfort	Yes	5 (20.0%)	0 (0.0%)	Not Applicable	
		No	20 (80.0%)	25 (100.0%)	Not Applicable	
ADR2	Skin Rash	Yes	0 (0.0%)	0 (0.0%)	Not Applicable	
		No	25 (100.0%)	25 (100.0%)	Not Applicable	
ADR3	Burning micturition	Yes	2 (8.0%)	0 (0.0%)	Not Applicable	
		No	23 (92.0%)	25 (100.0%)	Not Applicable	
ADR4	Excessive bleeding	Yes	0 (0.0%)	0 (0.0%)	Not Applicable	
		No	25 (100.0%)	25 (100.0%)	Not Applicable	
PO - Primary Outcome; SO - Secondary Outcome; ADR - Adverse drug reaction						
If 95% confidence interval for odds ratio contains value 1, then the parameter is not significant at 5% level.						
