# Supplementary material for: Oral Curcumin With Piperine as Adjuvant Therapy for the Treatment of COVID-19: A Randomized Clinical Trial
Source: Front Pharmacol. 2021 May 28;12:669362. doi: 10.3389/fphar.2021.669362 (PMC8193734; doi:10.3389/fphar.2021.669362)
Supplement: Supplementary file 3 [file Table3.pdf]

Supplementary Table 3: Severe subgroup: Statistical Analysis of Primary and Secondary outcomes.

Severe						
**Parameters**			**C3 Group**	**Non C3 Group**	**Odds Ratio (95% CI)**	**Significance**
Sample Size (n)			15	15		
**Sex**	Male		7 (46.7%)	11 (73.3%)		
	Female		8 (53.3%)	4 (26.7%)		
**Risk Factor**	Age >60		7 (46.7%)	5 (33.3%)	1.75 (0.40, 7.66)	Not Significant
	Unilateral pneumonia		0 (0.0%)	1 (6.7%)	Not Applicable	
	Bilateral pneumonia		15 (100.0%)	14 (93.3%)	Not Applicable	
Fever			12 (80.0%)	9 (60.0%)	2.67 (0.52, 13.66)	Not Significant
Cold			5 (33.3%)	3 (20.0%)	2.00 (0.38, 10.51)	Not Significant
Cough			11 (73.3%)	11 (73.3%)	1.00 (0.20, 5.05)	Not Significant
Sore throat			6 (40.0%)	1 (6.7%)	9.33 (0.96, 90.94)	Not Significant
Sneezing			1 (6.7%)	0 (0.0%)	Not Applicable	
Runny nose			1 (6.7%)	0 (0.0%)	Not Applicable	
Shortness of breath			6 (40.0%)	12 (80.0%)	0.17 (0.03, 0.85)	Significant
Headache			0 (0.0%)	1 (6.7%)	Not Applicable	
Diarrhoea			1 (6.7%)	0 (0.0%)	Not Applicable	
Myalgia			3 (20.0%)	5 (33.3%)	0.50 (0.10, 2.63)	Not Significant
Fatigue			3 (20.0%)	2 (13.3%)	1.63 (0.23, 11.46)	Not Significant
Loss of taste			0 (0.0%)	0 (0.0%)	Not Applicable	
Symptoms<6d			11 (73.3%)	11 (73.3%)	1.00 (0.20, 5.05)	Not Significant
Symptoms>6d			3 (20.0%)	4 (26.7%)	0.69 (0.12, 3.79)	Not Significant
**PO2**	Patient showed red flag signs	Yes	15 (100.0%)	15 (100.0%)	Not Applicable	
		No	0 (0.0%)	0 (0.0%)	Not Applicable	
**PO3**	Patient maintained SpO2 above 94% on room air throughout the stay	Yes	0 (0.0%)	0 (0.0%)	Not Applicable	
		No	14 (93.3%)	15 (100.0%)	Not Applicable	
**PO4**	Number of days patient could not maintain SpO2 above 94% on room air	No	0 (0.0%)	15 (100.0%)	Not Applicable	
**PO5**	Patient required Oxygen therapy	Yes	13 (86.7%)	15 (100.0%)	Not Applicable	
		No	2 (13.3%)	0 (0.0%)	Not Applicable	
**PO7**	Patient required Oxygen therapy with high flow nasal cannula (HFNC)	Yes	2 (13.3%)	11 (73.3%)	0.06 (0.01, 0.37)	Significant
		No	12 (80.0%)	4 (26.7%)	11.00 (2.00, 60.57)	Significant
**PO8**	Patient failed to maintain SpO2 more than 88 % despite of oxygenation	Yes	3 (20.0%)	8 (53.3%)	0.22 (0.04, 1.11)	Not Significant
		No	12 (80.0%)	7 (46.7%)	4.57 (0.90, 23.14)	Not Significant
**PO9**	intubation	Yes	1 (6.7%)	1 (6.7%)	1.00 (0.06, 17.62)	Not Significant
		No	9 (60.0%)	11 (73.3%)	0.55 (0.12, 2.55)	Not Significant
	mechanical ventilation (noninvasive / invasive)	MV-NI	1 (6.7%)	6 (40.0%)	0.11 (0.01, 1.04)	Not Significant
		MV-I	1 (6.7%)	0 (0.0%)	Not Applicable	
**PO10**	CRP rise		14 (93.3%)	15 (100.0%)	Not Applicable	
	CRP decline		0 (0.0%)	0 (0.0%)	Not Applicable	
	CRP approximately same		1 (6.7%)	0 (0.0%)	Not Applicable	
	D-Dimer rise		12 (80.0%)	15 (100.0%)	Not Applicable	
	D-Dimer decline		0 (0.0%)	0 (0.0%)	Not Applicable	
	D-Dimer approximately same		1 (6.7%)	0 (0.0%)	Not Applicable	
	N/L>3.5		9 (60.7%)	9 (60.0%)	1.00 (0.23, 4.31)	Not Significant
	N/L<3.5		4 (26.7%)	5 (33.3%)	0.73 (0.15, 3.49)	Not Significant
**PO11**	Chest X Ray abnormalities	Yes	15 (100.0%)	15 (100.0%)	Not Applicable	
		No	0 (0.0%)	0 (0.0%)	Not Applicable	
**PO12**	Patient required COVID CARP protocol	Yes	13 (86.7%)	14 (93.3%)	0.46 (0.04, 5.75)	Not Significant
		No	2 (13.3%)	1 (6.7%)	2.15 (0.17, 26.67)	Not Significant
**PO13**	Patient required LMW Heparin	Yes	12 (80.0%)	13 (86.7%)	0.62 (0.09, 4.34)	Not Significant
		No	3 (20.0%)	2 (13.3%)	1.63 (0.23, 11.46)	Not Significant
**PO14**	Patient required Remdesivir	Yes	13 (86.7%)	15 (100.0%)	Not Applicable	
		No	2 (13.3%)	0 (0.0%)	Not Applicable	
**PO15**	Patient required cytokine storm treatment eg. Tocilizumab	Yes	2 (13.3%)	4 (26.7%)	0.42 (0.06, 2.77)	Not Significant
		No	13 (86.7%)	11 (73.3%)	2.36 (0.36, 15.46)	Not Significant
**SO2**	Duration of hospitalisation	<10 days	12 (80.0%)	5 (33.3%)	8.00 (1.52, 42.04)	Significant
		10-14 day	2 (13.3%)	4 (26.7%)	0.42 (0.06, 2.77)	Not Significant
		>14days	1 (6.7%)	6 (40.0%)	0.11 (0.01, 1.04)	Not Significant
**SO3**	Thromboembolic events	Yes	0 (0.0%)	2 (13.3%)	Not Applicable	
		No	14 (93.3%)	13 (86.7%)	2.15 (0.17, 26.67)	Not Significant
	Pulmonary fibrosis	Yes	2 (13.3%)	14 (93.3%)	0.01 (0.00, 0.14)	Significant
		No	13 (86.7%)	1 (6.7%)	91.00 (7.35, 1126.95)	Significant
**SO4**	Death related to SARS CoV2 and causes of mortality	Yes	2 (13.3%)	5 (33.3%)	0.31 (0.05, 1.93)	Not Significant
		No	13 (86.7%)	8 (53.3%)	5.69 (0.94, 34.46)	Not Significant
**ADR1**	Nausea, Vomiting, Abdominal discomfort	Yes	0 (0.0%)	0 (0.0%)	Not Applicable	
		No	15 (100.0%)	15 (100.0%)	Not Applicable	
**ADR2**	Skin Rash	Yes	0 (0.0%)	0 (0.0%)	Not Applicable	
		No	15 (100.0%)	15 (100.0%)	Not Applicable	
**ADR3**	Burning micturition	Yes	1 (6.7%)	0 (0.0%)	Not Applicable	
		No	14 (93.3%)	15 (100.0%)	Not Applicable	
**ADR4**	Excessive bleeding	Yes	0 (0.0%)	0 (0.0%)	Not Applicable	
		No	15 (100.0%)	15 (100.0%)	Not Applicable	
PO - Primary Outcome; SO - Secondary Outcome; ADR - Adverse drug reaction						
If 95% confidence interval for odds ratio contains value 1, then the parameter is not significant at 5% level.						
